# Supplementary material for: Fibrinogen function achieved through multiple covalent states
Source: Nat Commun. 2020 Oct 29;11:5468. doi: 10.1038/s41467-020-19295-7 (PMC7596563; doi:10.1038/s41467-020-19295-7)
Supplement: Supplementary file 1 — Supplementary Information [file 41467_2020_19295_MOESM1_ESM.pdf]

SUPPLEMENTARY INFORMATION

Fibrinogen function achieved through multiple covalent states

Diego Butera and Philip J. Hogg

Supplementary Table 1. Fibrin(ogen) cysteine containing peptides analysed by HPLC and mass spectrometry. Cysteine numbering is according to UniProt identifiers P02671, P02675 and P02679 for human fibrinogen α, β and γ chains, respectively. The Cys residues of the disulfide that were measured are underlined in the peptide.

**Disulfide bond**	**Cys measured**	**Peptide**
αC47-αC47	αC47	HQSACK
αC55-βC95	αC55	DSDWPFCSDEDWNYK
αC64-γC49	αC64	CPSGCR
	γC49	FGSYCPTTCGIADFLSTYQTK
αC68-βC106	αC68	CPSGCR
βC110-γC45	γC45	FGSYCPTTCGIADFLSTYQTK
αC180-γC161	γC161	VAQLEAQCQEPCK
αC184-βC223	βC223	LESDVSAQMEYCR
αC461-αC491	αC491	EVVTSEDGSDCPEAMDLGTLSGIGTLDGFR
βC227-γC165	βC227<br>γC165	TPCTVSCNIPVVSGK<br>VAQLEAQCQEPCK
βC231-βC316	βC231<br>βC316	TPCTVSCNIPVVSGK<br>NYCGLPGEYWLGNDK
βC241-βC270	βC241	ECEEIIR
	βC270	VYCDMNTENGGWTVIQNR
βC424-βC437	βC437	CHAANPNGR
γC179-γC208	γC179	DCQDIANK
	γC208	ANQQFLVYCEIDGSGNGWTVFQK
γC352-γC365	γC352	FEGNCAEQDGSGWWMNK
	γC365	CHAGHLNGVYYQGGTYSK

Supplementary Table 2. Healthy donor fibrinogen was immunoprecipitated from plasma, digested with trypsin, peptides resolved by HPLC and analysed by mass spectrometry. Disulfide-linked peptides were identified using Byonic analysis software. The cysteines involved in the disulfide linkage are underlined.

Disulfide bond	Peptide 1	Peptide 2
αC47-αC47	HQSACK	HQSACK
αC184-βC223	GSCSR	LESDVSAQMEYCR
	GSCSR	LESDVSAQM(Ox)EYCR
αC180-γC161	SCR	VAQLEAQCQEPC(^{12}C-IPA)K
αC461-αC491	RSCSK	EVVTSEDGSDCPEAMDLGTLSGIGTLDGFR
	SCSK	EVVTSEDGSDCPEAMDLGTLSGIGTLDGFR
	RSCSK	EVVTSEDGSDCPEAM(Ox)DLGTLSGIGTLDGFR
	SCSK	EVVTSEDGSDCPEAM(Ox)DLGTLSGIGTLDGFR
βC241-βC270	ECEEIIR	VYCDMNTENGGWTVIQNR
	ECEEIIR	VYCDM(Ox)NTENGGWTVIQNR
βC424-βC437	KQCSK	CHAANPNGR
	QCSK	CHAANPNGR
γC179-γC208	DCQDIANK	ANQQFLVYCEIDGSGNGWTVFQK

Supplementary Table 3. Redox states of the five E region and eight D region disulfides in fibrinogen (10 donors) and fibrin polymer (in 4 of the 10 donors). Statistics of the results shown in Fig. 2B. Discovery determined using the two-stage linear step-up procedure of Benjamini, Krieger and Yekutieli, with Q = 1%. Each row was analyzed individually, without assuming a consistent SD.

Disulfide bond	Fibrinogen mean % reduced	Fibrin mean % reduced	Difference ± SE	P value
αC47-αC47	17.83	12.03	5.80 ± 2.03	0.028721
αC55-βC95	18.30	11.36	6.94 ± 1.48	0.003349
αC64-γC49	24.99	15.80	9.18 ± 1.29	0.000392
αC68-βC106	31.69	24.15	7.55 ± 2.03	0.009795
βC110-γC45	23.96	14.34	9.62 ± 1.76	0.001583
αC180-γC161	13.56	8.05	5.52 ± 0.65	0.000149
αC184-βC223	31.14	18.26	12.88 ± 1.40	0.000094
βC227-γC165	22.80	18.11	4.70 ± 0.83	0.001286
βC231-βC316	31.86	19.00	12.86 ± 1.06	0.000019
βC241-βC270	29.28	20.19	9.09 ± 2.41	0.009284
βC424-βC437	53.87	34.73	19.14 ± 1.03	0.000002
γC179-γC208	9.50	5.13	4.37 ± 0.85	0.002066
γC352-γC365	40.10	24.58	15.52 ± 1.20	0.000013

Supplementary Table 4. α2-Macroglobulin cysteine containing peptides analysed by HPLC and mass spectrometry. Cysteine numbering is according to UniProt identifier P01023. The Cys residues of the disulfide that were measured are underlined in the peptide.

Disulfide bond	Cys measured	Peptide
C48-C86	C86	SLFTDLEAENDVLHCVAFAVPK
C251-C299	C299	FSGQLNSHGCFYQQVK
C269-C287	C287	KYSDASDCHGEDSQAFCEK
C278-C431	C278	KYSDASDCHGEDSQAFCEK
C470-C563	C470	SFVHLEPMSHELPCGHTQTVQAHYILNGGTLLGLK
	C563	YDVENCLANK
C595-C771	C595	VTAAPQSVCALR
	C771	AGAFCLSEDAGLGISSTASLR
C642-C689	C689	MCPQLQQYEMHGPEGLR
C821-C849	C849	EQAPHCICANGR
C847-C883	C847	EQAPHCICANGR
C921-C1321	C921	ETTFNSLLCPSGGEVSEELSLK
	C1321	VTGEGCVYLQTSLK
C1079-C1127	C1079	DNGCFR
	C1127	NALFCLESAWK
C1352-C1467	C1352	YNILPEKEEFPFALGVQTLPQTCDEPK
	C1467	VYDYYETDEFAIAEYNAPCSK

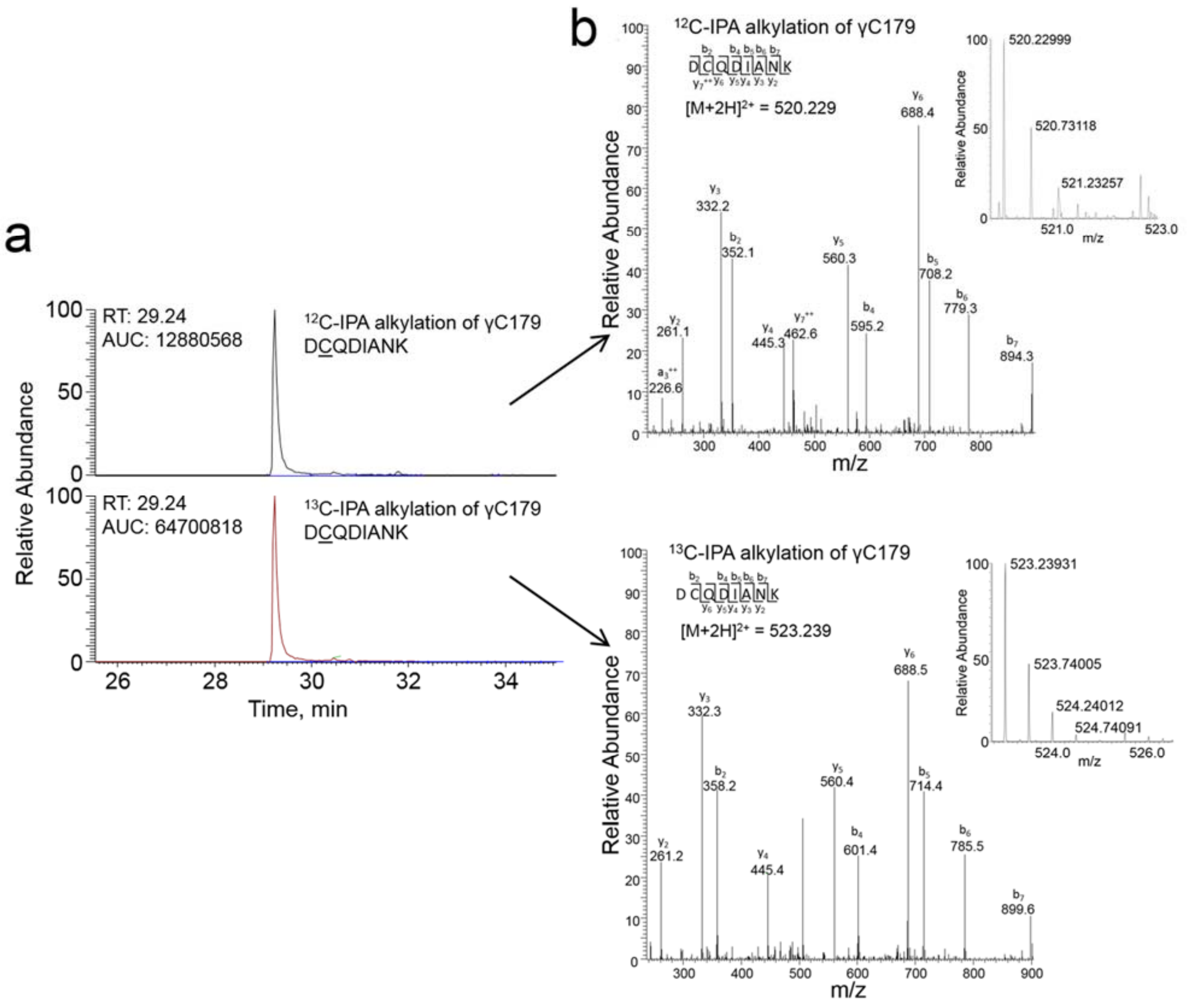

Supplementary Figure 1. Differential cysteine alkylation of the fibrinogen γ chain Cys179 residue and peptide analysis. **a**. HPLC resolution of the γ chain DCQDIANK peptide containing Cys179 labelled with either ^{12}C-IPA (upper trace) or ^{13}C-IPA (lower trace). **b**. Representative tandem mass spectra of the γ chain DCQDIANK peptide. The upper and lower traces are examples of ^{12}C-IPA or ^{13}C-IPA alkylation of Cys179, respectively. The accurate mass spectrum of the peptide is shown in the insets (upper trace, observed $[M+2H]^{2+}$ = 520.229 m/z and expected $[M+2H]^{2+}$ = 520.229 m/z; lower trace, observed $[M+2H]^{3+}$ = 523.239 m/z and expected $[M+2H]^{2+}$ = 523.239).

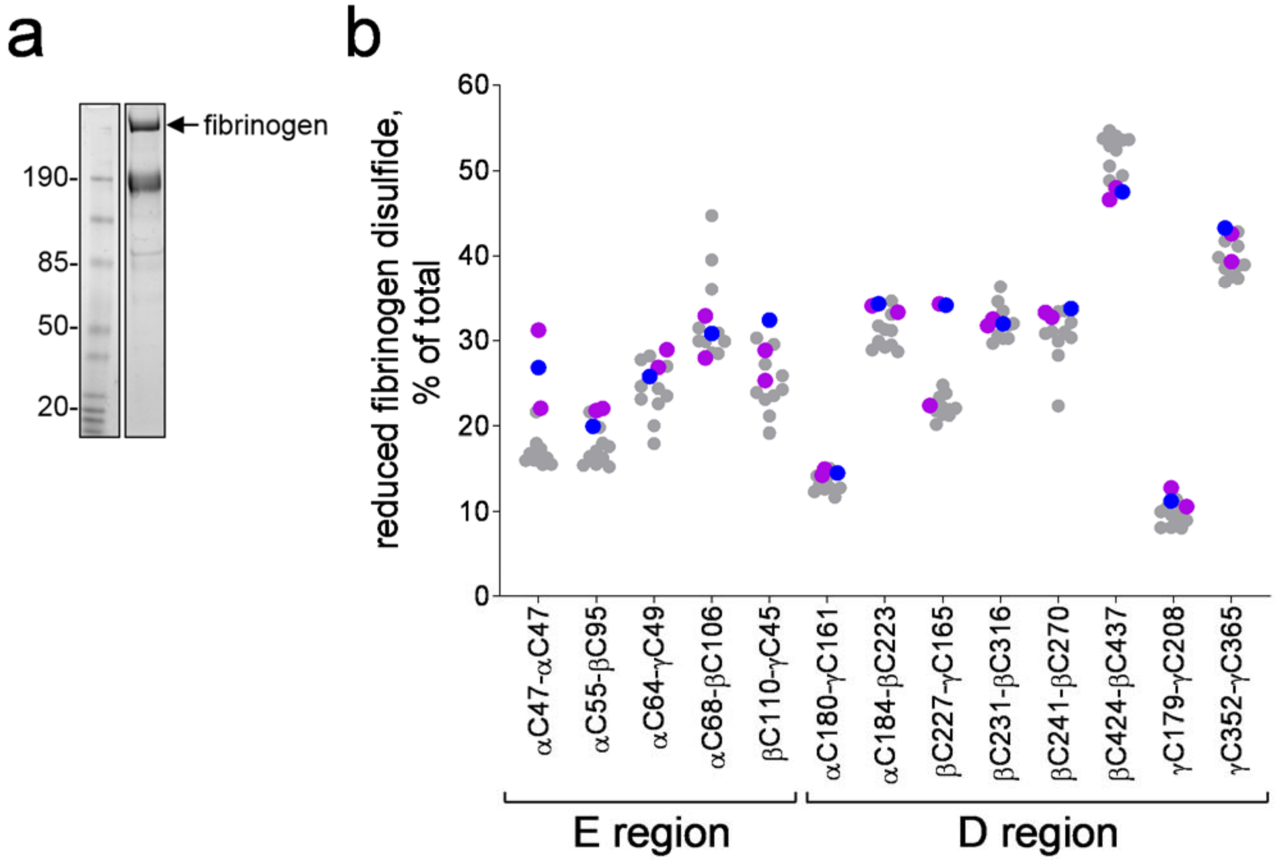

Supplementary Figure 2. The covalent states of fibrinogen secreted by cultured hepatocytes are indistinguishable from healthy donor plasma fibrinogen. **a**. Example of ^{12}C-IPA-labelled fibrinogen resolved on SDS-PAGE. Molecular mass standards are shown in the left-hand lane. **b**. Redox states of the five E region and eight D region disulfides from two biological replicates of HepG2 fibrinogen produced under standard normoxic (18.8% O_2) conditions (purple symbols) or a single experiment performed under hypoxic (1% O_2) conditions (blue symbols). The redox states of 10 healthy donor fibrinogens are shown for reference (grey symbols). Source data are provided as a Source Data file.

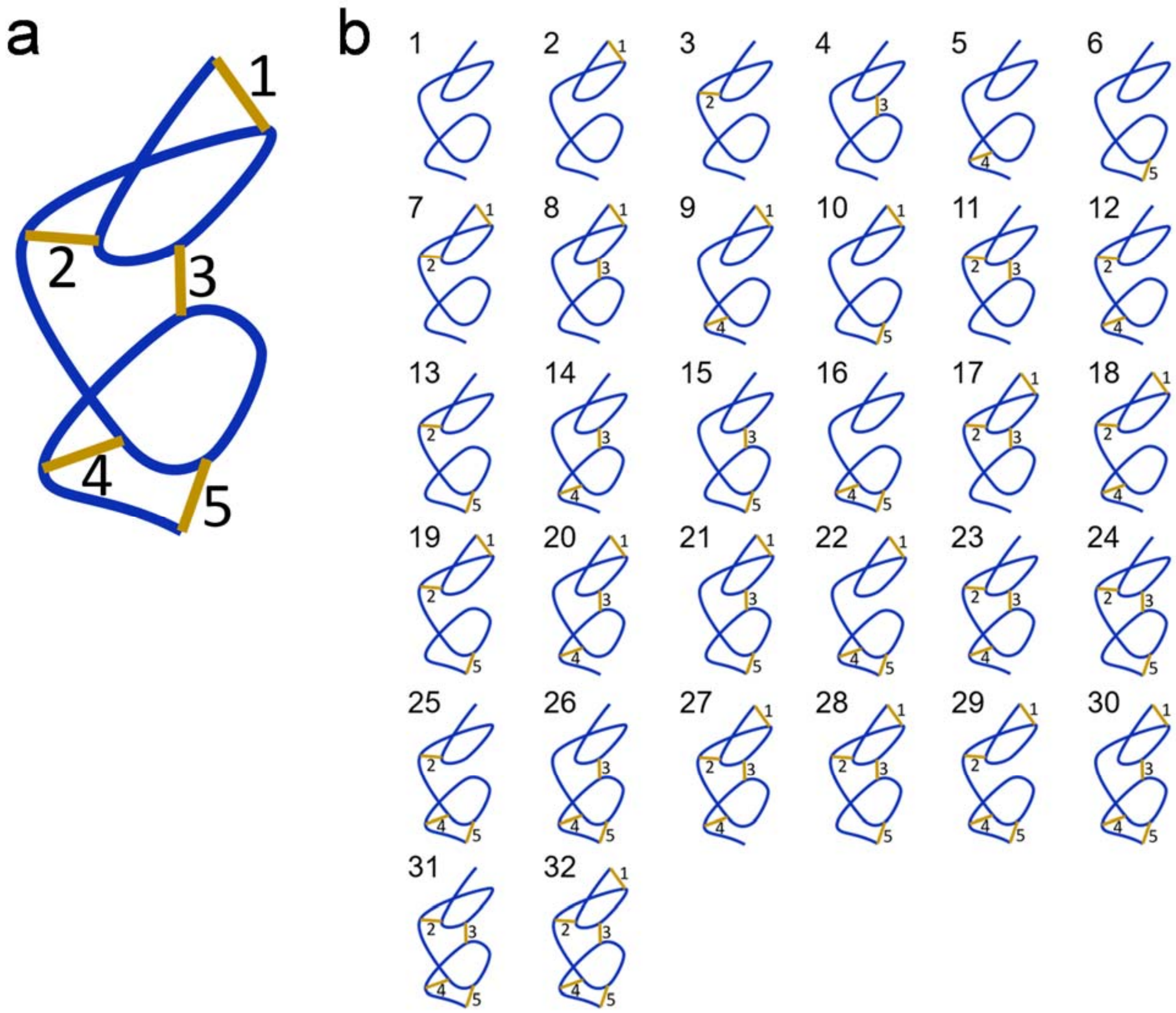

Supplementary Figure 3. A protein containing n disulfide bonds has 2^n possible disulfide-bonded states. **a**. A cartoon polypeptide containing 5 disulfide bonds. **b.** A polypeptide containing 5 disulfide bonds, where the bonds are either formed or broken, can exist in 32 possible disulfide states.

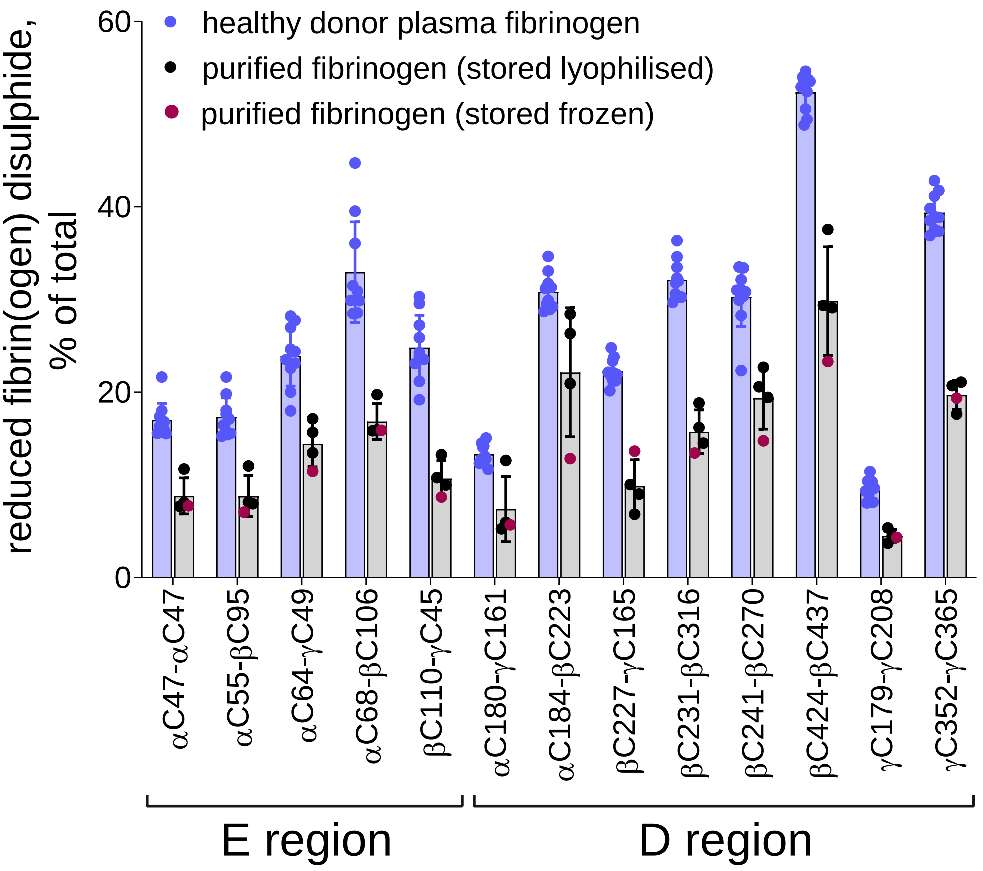

Supplementary Figure 4. Isolation of fibrinogen from plasma results in partial oxidation of the disulfide bonds. Redox states of the five E region and eight D region disulfides in healthy donor fibrinogen (n = 10) versus a lyophilised preparation of fibrinogen obtained commercially (n = 3, black symbols) or fibrinogen purified from healthy donor fresh frozen plasma by β-alanine precipitation and stored at -20°C (red symbols). The bars and errors are mean ± SD. All disulfides are significantly more oxidized in the purified fibrinogens ($p<0.01$). Parametric unpaired t test was used to evaluate differences between groups. Source data are provided as a Source Data file.

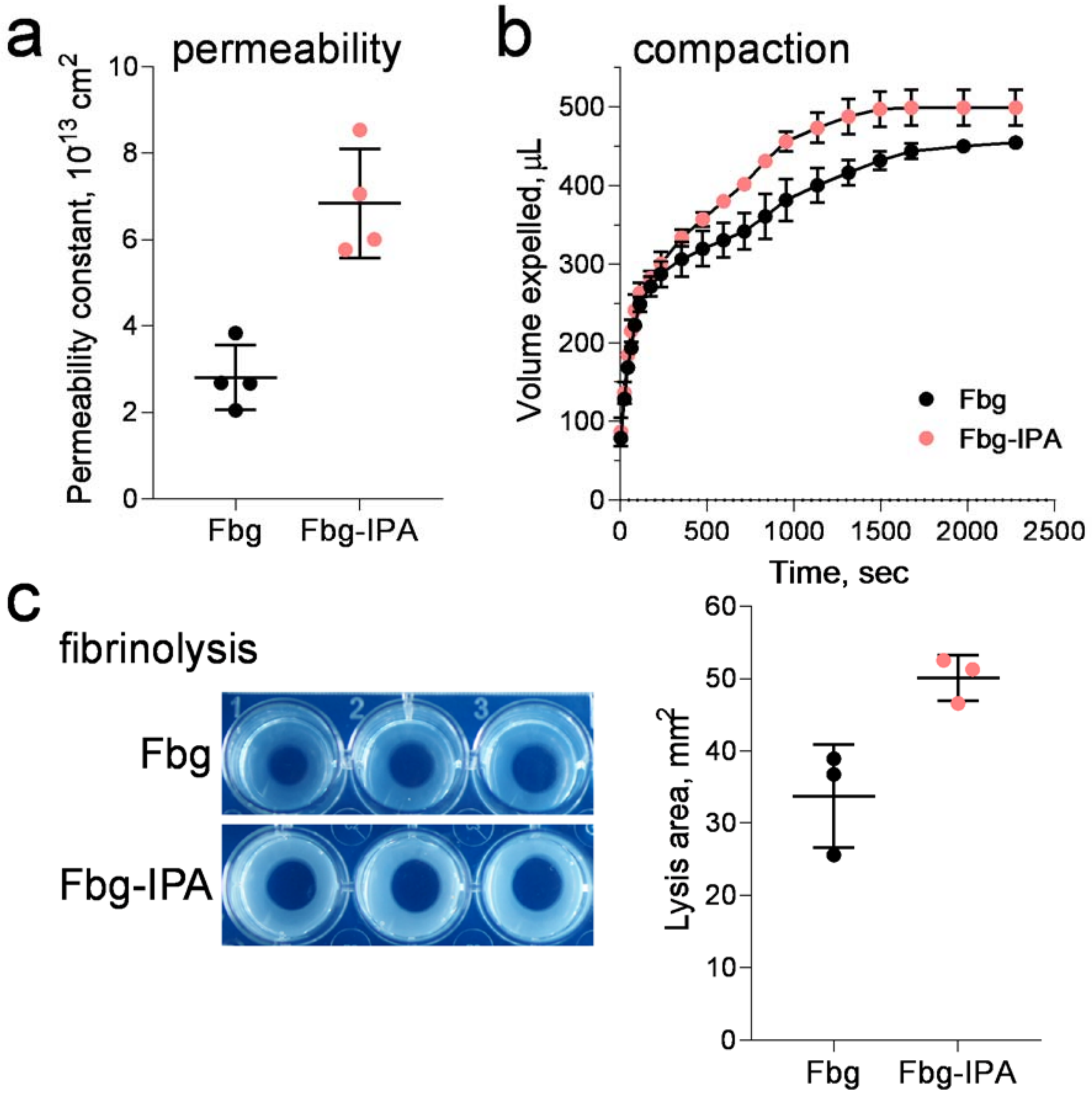

Supplementary Figure 5. Prevention of disulfide bond formation in fibrin polymer results in a more permeable and fragile matrix and one that is more readily lysed by plasmin. **a**. Permeation of fibrin polymer gels. Fibrin polymers were formed in chromatography columns, overlayed with 4 column volumes of buffer and the rate of permeation of the buffer measured. The permeation (Darcy) constants are from 4 independent experiments and the errors are mean ± SD. Source data are provided as a Source Data file. **b**. Kinetics of compaction of fibrin polymer gels. Fibrin polymers were subjected to centripetal force for discrete times and the volume of the fluid extruded from the polymer measured. The data points are the mean ± SD from 3 independent experiments. Source data are provided as a Source Data file. **c**. Plasmin lysis of fibrin polymers. Fibrin polymers were formed in 24-well plates, an aliquot of plasmin added to the centre of the wells and lysis areas determined from the cleared zones (figure at left). The data points are from 3 independent experiments (graph at right) and the errors are mean ± SD. Source data are provided as a Source Data file.

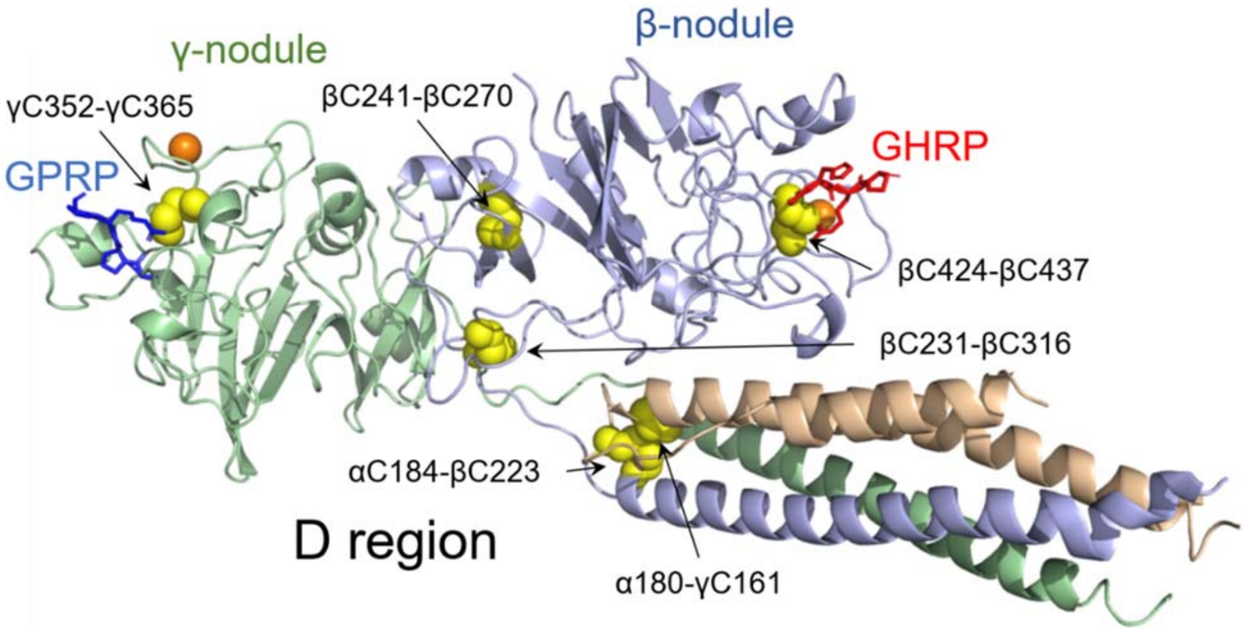

Supplementary Figure 6. Ribbon structure of the fibrin D region with peptide mimetics (sticks) bound in the 'a' (GPRP) and 'b' (GHRP) holes [1] (PDB identifier 1fzc). The α chains are in wheat, β chains in light blue and γ chains in light green. The positions of 6 disulfide bonds (yellow spheres) that are significantly more oxidized in fibrin than in fibrinogen are indicated by yellow spheres. Bound calcium ions are shown as orange balls.

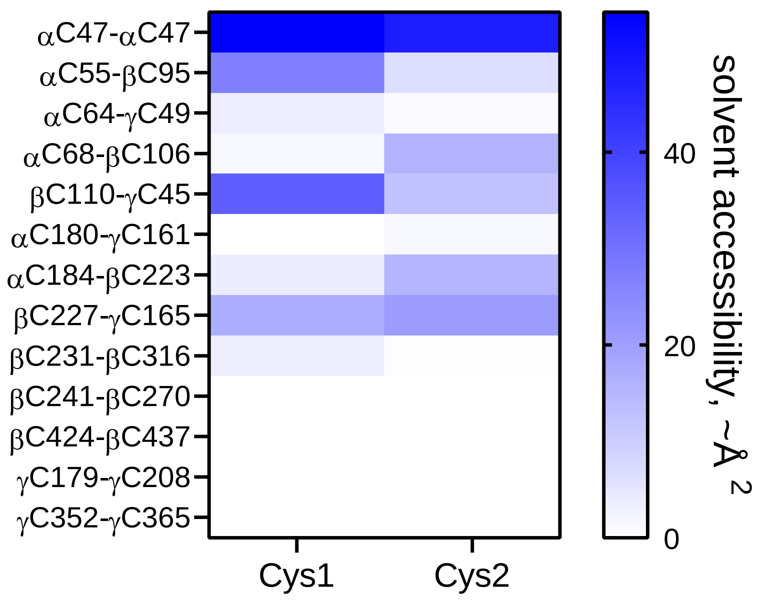

Supplementary Figure 7. Heat map of the solvent accessibility of the 13 fibrinogen disulfide bond cysteines. The scale is the solvent accessibility in ~$Å^2$ derived from DSSP[2] using the PDB identifier 3GHG structure of fibrinogen[3]. Source data are provided as a Source Data file.

Supplementary References

1. Everse SJ, Spraggon G, Veerapandian L, Riley M, Doolittle RF. Crystal structure of fragment double-D from human fibrin with two different bound ligands. *Biochemistry* **37**, 8637-8642 (1998).
2. Kabsch W, Sander C. Dictionary of protein secondary structure: pattern recognition of hydrogen-bonded and geometrical features. *Biopolymers* **22**, 2577-2637 (1983).
3. Kollman JM, Pandi L, Sawaya MR, Riley M, Doolittle RF. Crystal structure of human fibrinogen. *Biochemistry* **48**, 3877-3886 (2009).
