# Supplementary material for: Fibrinogen function achieved through multiple covalent states
Source: Nat Commun. 2020 Oct 29;11:5468. doi: 10.1038/s41467-020-19295-7 (PMC7596563; doi:10.1038/s41467-020-19295-7)
Supplement: Supplementary file 3 — Description of Additional Supplementary Files [file 41467_2020_19295_MOESM3_ESM.docx]

File Name: Supplementary Data 1
Description: HPLC retention times of fibrinogen peptides and ^12^C-IPA- and ^13^C-IPA-labelled peptide AUC values for the ten healthy donor proteins. Values for peptides containing a single Cys are listed. For peptides containing two Cys we obtain 3 populations: one where both Cys are labelled with ^12^C-IPA, one where both Cys are labelled with ^13^C-IPA, and a mixed population where one Cys is labelled with ^12^C-IPA and the other with ^13^C-IPA at either position. The mixed populations have the same retention time and mass. To distinguish between them, we select fragments from MS2 that have unique mass signatures for one or the other. Since each mass has intensity values we can compare the total intensities for both peptides and find their ratio. αC491 is predicted to pair with αC461, while the cysteine that pairs with αC663 is not known.

File Name: Supplementary Data 2
Description: HPLC retention times of α2-macroglobulin peptides and ^12^C-IPA- and ^13^C-IPA-labelled peptide peak AUC values for the eight healthy donor proteins. Values for peptides containing a single Cys are listed.
