# Supplementary material for: Fibrinogen function achieved through multiple covalent states
Source: Nat Commun. 2020 Oct 29;11:5468. doi: 10.1038/s41467-020-19295-7 (PMC7596563; doi:10.1038/s41467-020-19295-7)
Supplement: Supplementary file 6 — Reporting Summary [file 41467_2020_19295_MOESM6_ESM.pdf]

nature research

Corresponding author(s): Philip Hogg

Last updated by author(s): Sep 30, 2020

# Reporting Summary

Nature Research wishes to improve the reproducibility of the work that we publish. This form provides structure for consistency and transparency in reporting. For further information on Nature Research policies, see our Editorial Policies and the Editorial Policy Checklist.

## Statistics

For all statistical analyses, confirm that the following items are present in the figure legend, table legend, main text, or Methods section.

| n/a | Confirmed | |
|---|---|---|
| ☐ | ☒ | The exact sample size ($n$) for each experimental group/condition, given as a discrete number and unit of measurement |
| ☐ | ☒ | A statement on whether measurements were taken from distinct samples or whether the same sample was measured repeatedly |
| ☐ | ☒ | The statistical test(s) used AND whether they are one- or two-sided<br>*Only common tests should be described solely by name; describe more complex techniques in the Methods section.* |
| ☒ | ☐ | A description of all covariates tested |
| ☒ | ☐ | A description of any assumptions or corrections, such as tests of normality and adjustment for multiple comparisons |
| ☐ | ☒ | A full description of the statistical parameters including central tendency (e.g. means) or other basic estimates (e.g. regression coefficient) AND variation (e.g. standard deviation) or associated estimates of uncertainty (e.g. confidence intervals) |
| ☐ | ☒ | For null hypothesis testing, the test statistic (e.g. $F$, $t$, $r$) with confidence intervals, effect sizes, degrees of freedom and $P$ value noted<br>*Give P values as exact values whenever suitable.* |
| ☒ | ☐ | For Bayesian analysis, information on the choice of priors and Markov chain Monte Carlo settings |
| ☒ | ☐ | For hierarchical and complex designs, identification of the appropriate level for tests and full reporting of outcomes |
| ☒ | ☐ | Estimates of effect sizes (e.g. Cohen's $d$, Pearson's $r$), indicating how they were calculated |

*Our web collection on statistics for biologists contains articles on many of the points above.*

## Software and code

Policy information about availability of computer code

| | |
|---|---|
| Data collection | No software was used for data collection |
| Data analysis | Mascot Daemon Version 2.5.0; MS-Product v 6.2.2; XCalibur Qual Browser v2.1.0; Byonic Version 3.9-7; ImageJ Version 1.52a; DSSP Version 3 |

For manuscripts utilizing custom algorithms or software that are central to the research but not yet described in published literature, software must be made available to editors and reviewers. We strongly encourage code deposition in a community repository (e.g. GitHub). See the Nature Research guidelines for submitting code & software for further information.

## Data

Policy information about availability of data

All manuscripts must include a data availability statement. This statement should provide the following information, where applicable:

- Accession codes, unique identifiers, or web links for publicly available datasets
- A list of figures that have associated raw data
- A description of any restrictions on data availability

The mass spectrometry proteomics data have been deposited to the ProteomeXchange Consortium via the PRIDE [1] partner repository with the dataset identifier PXD018564.
PDB codes: 3ghg, 4acq
Figures that have associated raw data: Figure 1d, Figure 2b, Figure 3a, Figure 3b, Figure 4b, Figure 4c, Figure 5a, Figure 5b, Figure 6b, Supplementary Figure 2b, Supplementary Figure 4, Supplementary Figure 5a, Supplementary Figure 5b, Supplementary Figure 5c, Supplementary Figure 7
All other relevant data are available from the authors.

# Field-specific reporting

Please select the one below that is the best fit for your research. If you are not sure, read the appropriate sections before making your selection.

[x] Life sciences  [ ] Behavioural & social sciences  [ ] Ecological, evolutionary & environmental sciences

For a reference copy of the document with all sections, see nature.com/documents/nr-reporting-summary-flat.pdf

# Life sciences study design

All studies must disclose on these points even when the disclosure is negative.

| | |
|---|---|
| Sample size | No sample size calculation was performed. 2-10 biological replicates were performed for each experiment. Number of replicates were chosen based on the variability in the data set. For example, 13 disulfide bonds exist in bound or cleaved forms in the fibrinogen populations of ten healthy human donors (4 male, 6 female, 22-58 years old). The bonds ranged from 10 to 50% reduced and there was remarkably small donor-to-donor variation and no significant gender difference. The coefficients of variation ranged from a low of 3.9% for the βC424-βC437 disulfide to a high of 16.5% for the αC68-βC106 bond. |
| Data exclusions | We did not exclude any relevant data |
| Replication | All experiments have been performed on more than one occasion and in some cases by different investigators. All attempts at replication were successful, although there were minor quantitative variations from experiment to experiment. |
| Randomization | Randomization was not applicable to the study. Healthy donor and ECMO patient plasmas were collected as donors or patients became available. There was no selection of donor/patient samples other than to ensure both males and females of different ages were included. |
| Blinding | Blinding was not applicable to the study. Healthy donor and ECMO patient plasmas were collected as donors or patients became available. There was no selection of donor/patient samples other than to ensure both males and females of different ages were included. |

# Reporting for specific materials, systems and methods

We require information from authors about some types of materials, experimental systems and methods used in many studies. Here, indicate whether each material, system or method listed is relevant to your study. If you are not sure if a list item applies to your research, read the appropriate section before selecting a response.

## Materials & experimental systems

| n/a | Involved in the study | |
|---|---|---|
| [ ] | [x] | Antibodies |
| [ ] | [x] | Eukaryotic cell lines |
| [x] | [ ] | Palaeontology and archaeology |
| [x] | [ ] | Animals and other organisms |
| [ ] | [x] | Human research participants |
| [x] | [ ] | Clinical data |
| [x] | [ ] | Dual use research of concern |

## Methods

| n/a | Involved in the study | |
|---|---|---|
| [x] | [ ] | ChIP-seq |
| [x] | [ ] | Flow cytometry |
| [x] | [ ] | MRI-based neuroimaging |

## Antibodies

| | |
|---|---|
| Antibodies used | Polyclonal anti-fibrinogen (Cat A0080, Lot 00015063) and anti-α2-macroglobulin (Cat Q0102, Lot 20068567) antibodies from Dako. |
| Validation | Immunoprecipitated fibrinogen and anti-α2-macroglobulin were validated extensively by mass spectrometry in the study. |

## Eukaryotic cell lines

Policy information about cell lines

| | |
|---|---|
| Cell line source(s) | American Type Culture Collection, HepG2, HB-8065 |
| Authentication | The cell line was not authenticated, other than it constitutively secreted human fibrinogen. |
| Mycoplasma contamination | This was tested and not found. |
| Commonly misidentified lines (See ICLAC register) | Nil |

# Human research participants

Policy information about studies involving human research participants

| | |
|---|---|
| Population characteristics | Plasma from ten healthy human donors (4 male, 6 female, 22-58 years old) and eight patients at a single center who had ECMO support (4 male, 4 female, 34-67 years old) was employed in the study. Patients with ECMO support received anti-coagulation and/or anti-platelet medications based on at clinicians' discretion or institutional guideline where patients typically commenced on warfarin, target international normalized ratio (INR) 2-3, with bridging heparin infusion and aspirin therapy, as well as dipyridamole for those who are considered high risk for thrombosis. |
| Recruitment | Healthy donor and ECMO patient plasmas were collected as donors or patients became available. There was no selection of donor/patient samples other than to ensure both males and females of different ages were included. |
| Ethics oversight | All procedures involving collection of human blood from healthy volunteers were in accordance with the Human Research Ethics Committee of the University of Sydney (approval HREC 2014/244) and informed consent was obtained from all individuals. All procedures involving collection of human blood from ECMO patients were in accordance with the Alfred Hospital Ethics, Monash University Standing Committee for Research in Humans (approval 388/13) and informed consent was obtained from all individuals. All procedures were in accordance with the Helsinki Declaration of 1983. |

Note that full information on the approval of the study protocol must also be provided in the manuscript.
